# The Action System Model: A Typology of Spanish Homicides

**DOI:** 10.3389/fpsyg.2020.585279

**Published:** 2020-09-16

**Authors:** María del Mar Pecino-Latorre, Jorge Santos-Hermoso, María del Carmen Pérez-Fuentes, Rosa María Patró-Hernández, José Luis González Álvarez

**Affiliations:** ^1^Department of Psychology, University of Almería, Almería, Spain; ^2^Department of Biological and Health Psychology, Faculty of Psychology, Autonomous University of Madrid, Madrid, Spain; ^3^Department of Psychology, Faculty of Psychology, Universidad Autónoma de Chile, Santiago, Chile; ^4^Department of Psychology, University of Murcia, Murcia, Spain; ^5^Cabinet of Coordination and Studies of the Secretary of State for Security, Spanish Ministry of Interior, Madrid, Spain

**Keywords:** action system, homicides, investigative psychology, crime scene behavior, multidimensional scaling, cluster analysis

## Abstract

The Action System model offers a scientific foundation to the differentiation and classification of crimes, based on behavioral indicators, allowing the establishment of relationships between the actions carried out by the offender on the crime scene and their characteristics. Although it was originally developed for application to fires, its utility has been tested in distinct criminal typologies, with few studies having considered homicides. The objective of this study was to examine the effectiveness and validity of the Action System model to differentiate thematically between the structure of the homicides and to create a typology of simple homicides in Spain, based on the relationships between the *modus operandi*, characteristics of the victims and characteristics of the offenders. The sample consisted of 448 homicides. Four homicide typologies were identified: Expressive, Adaptive, Integrative and Conservative, which represent 87.5% of the studied cases. Expressive homicides are impulsive, with offenders having criminal records and previously knowing their victims. Adaptive homicides are linked to robberies and sexual aggressions, in which the victim and offender are strangers. Integrative homicides take place in the family environment, specifically female offenders and femicides. Conservative homicides are very well planned, highlighting the presence of post mortem actions. The findings of this work suggest that the Action System model is a useful theoretical framework for the identification of variations in criminal behavior and understanding of the psychological processes underlying the homicides. These results have practical implications in the academic setting, since they offer a global perspective as to how simple homicides in Spain may be differentiated, also within the framework of criminal profiling, specifically, suspect prioritization.

## Introduction

Homicides are considered to be the most extreme manifestation of criminal behavior (Liem, 2013). The International Classification of Crime defines it as “*unlawful death inflicted upon a person with the intent to cause death or serious injury*” (United Nations Office on Drugs and Crime [UNODC], 2015, 17). Over recent decades, its study has been of great interest in the academic and professional areas, not only due to the seriousness of the offense, but also given its repercussions on psychosocial, political and economic aspects of a country (Botelho and Gonçalves, 2016; Vichi et al., 2020). And, homicides promote fear, social alarm and the perception of insecurity in a country, influencing the assessment made by society on the effectiveness of its police forces (Ganpat et al., 2011; Braga and Dusseault, 2018; González et al., 2018).

At the global level, homicides rates are relatively stable or declining for over the last few decades (Liem and Pridemore, 2012; United Nations Office on Drugs and Crime [UNODC], 2019). The global average homicide rate in 2017 was reported to be 6.1 per 100,000 population, with the highest rates was recorded in Central America and South America. By contrast, the subregions with the lowest levels of homicide were Southern, Western and Northern Europe (Weiss et al., 2016; United Nations Office on Drugs and Crime [UNODC], 2019; Miles and Buehler, 2020). Currently, Spain is one of the European countries with the lowest homicide rates (0.6), whereas the United States and the United Kingdom where the majority of homicide research is conducted, have rates higher than that (5 and 1.2, respectively) (United Nations Office on Drugs and Crime [UNODC], 2018).

Of the current trends in homicide studies, some works have focused on prediction, given the major practical implications that it has on police investigations (González-Álvarez et al., 2015; Abreu et al., 2018; Fox and Farrington, 2018). Specifically, there has been an interest in examining the utility of criminal profiling, as a criminal investigation technique directed at assisting in the prioritization and detention of offenders (Chan et al., 2019; Pecino-Latorre et al., 2019a; Sorochinski and Salfati, 2019; Ivaskevics and Almond, 2020). Therefore, numerous typologies have been created for homicides, always based on empirical evidence to ensure a more rigorous and systematic profile creation process (Horning et al., 2015; Fujita et al., 2016; Khoshnood and Väfors Fritz, 2017; Sea et al., 2017).

Similarly, many research studies have established a homicide classification, based on the Instrumental/Expressive dichotomy of aggressive behavior (Salfati and Park, 2007; Goodwill et al., 2014; Adjorlolo and Chan, 2015; Sea and Beauregard, 2017; Pecino-Latorre et al., 2019b). However, some authors consider that the Instrumental/Expressive instrument is an excessive simplification of the violence as it attributes the behavior to only one psychological construct (Häkkänen et al., 2004b; Thijssen and De Ruiter, 2011). Therefore, Canter and Youngs (2009) offer more complex models of criminal differentiation such as the Action System model, based on the General Systems Theory (von Bertalanffy, 1968; Shye, 1985).

Based on the Action System model it is assumed that criminal behavior includes two main facets, the source of the criminal action and the desired target of the criminal behavior, which may be internal or external to the offender (Canter and Fritzon, 1998). Thus, offenders can be differentiated based on whether the motivating source of their crime is expressive (internal) or instrumental (external) in nature (Fritzon et al., 2014). Similarly, offenders can also be categorized based on the nature of the target and whether it is external (object) or internal (person) to the offender. Furthermore, previous studies has suggested that offenders could be classified based not only on their crime scene actions but by their background characteristics too (Fritzon et al., 2001; Häkkänen et al., 2004b). In this way, the combination of these two facets leads to four modes of functioning: Expressive, Adaptive, Integrative and Conservative.

In the Expressive mode, the offender perceives the victim as an object on which his/her feelings of anger and frustration may be manifested (Canter, 2010; Hollows and Fritzon, 2012). Homicides included in this mode reflect a great impulsivity, with the main trigger being interpersonal conflicts between the victim and the author (Fritzon and Brun, 2005; Cullen and Fritzon, 2019). Also, it is likely that this type of violence is common in offenders who experience a negative emotional state; therefore, it is characteristic of offenders with criminal records (Fritzon and Brun, 2005; Cullen and Fritzon, 2019). In the Adaptive mode, the actions have an instrumental nature, and violence is a means of achieving the desired objectives of the offender (e.g., economic or sexual benefits) (Fritzon and Brun, 2005). In these cases, the victims do not hold a special significance to the offender, since they are not killed for who they are, but rather, for what they represent (Fritzon and Garbutt, 2001; Fritzon and Brun, 2005). In the Integrative mode, homicides are related to an emotional explosion, interpreted as a form of freeing up accumulated emotional tension of the offender and, in the most extreme cases, the offender may commit suicide after carrying out the homicide (Fritzon and Garbutt, 2001; Fritzon and Brun, 2005; Cullen and Fritzon, 2019). In these homicides, it is likely that the victim will be from the family setting (Fritzon and Garbutt, 2001; Cullen and Fritzon, 2019) and the offender will suffer from some sort of emotional disorder (Fritzon, 2013). In the Conservative mode, the actions carried out by the offender are not emotional, but rather, instrumental, as an attempt is made to maintain an integral part of his/her personality that has been attacked (e.g., cultural values, religious beliefs) (Fritzon and Brun, 2005). This is characteristic of homicides that are precipitated by arguments and are motivated by a desire for revenge, control and power; in addition, it is characterized by much criminal planning, given the large degree of psychological rumination (Fritzon and Garbutt, 2001; Fritzon and Brun, 2005; Fritzon, 2013).

This model not only identifies four means of functioning, but also establishes similarities and differences between them (Fritzon et al., 2001; Fritzon, 2013). Considering these relationships, their disposition in the multi-dimensional space responds to a specific configuration. Therefore, the Expressive and Conservative modes appear to conflict with one another, being significantly different in terms of origin of the action, as is the case with the Integrative and Adaptive modes, in terms of objective of the action. Similarly, the direction of the latter is perpendicular to the direction outlined by the Expressive and Conservative modes (Canter, 2010).

Therefore, the Action System model offers a scientific foundation to differentiate and classify crimes based on behavioral indicators, allowing for the development of relationships between the actions carried out by offenders on the crime scene and their characteristics (Canter and Youngs, 2009; Canter, 2010). Similarly, it permits inferences to be made between certain psychological characteristics and characteristics of personality that are associated with distinct forms of crime, facilitating the understanding of the psychological processes underlying the crime (Canter and Fritzon, 1998; Fritzon, 2013; Cullen and Fritzon, 2019).

Although this model was initially used to identify variations in criminal activity and determine criminological profiles in fires (Canter and Fritzon, 1998; Fritzon et al., 2001, 2014; Santtila et al., 2003; Häkkänen et al., 2004b; Almond et al., 2005), later, some authors have demonstrated its use in distinct crime typologies, such as terrorism (Fritzon et al., 2001), critical incidents (Hempenstall and Hammond, 2018), genocide (Hollows and Fritzon, 2012) and rape (Canter et al., 2003; Häkkänen et al., 2004a). In the case of homicide, few studies have explored its use, and the majority of those that have considered it have focused on a specific type, such as familicide (Fritzon and Garbutt, 2001; Cullen and Fritzon, 2019) and homicides taking place in the school setting (Fritzon and Brun, 2005).

After conducting a scientific literature review, no prior studies from Spain were found that used this model to classify homicides into typologies and link them to characteristics of the offenders. Therefore, the objective of this work is to verify the effectiveness and validity of the Action System model in order to offer a thematic differentiation of the homicide structure and create a typology of simple homicides in Spain based on the relationships between the *modus operandi*, victim characteristics and offender characteristics.

Specifically, four modes of functioning were identified: Expressive, Conservative, Integrative and Adaptive, which lead to a typology of simple homicides, according to the theoretical foundations of the Action System model.

Similarly, it is expected that each of the cases studied will be classified in a dominant functioning mode, demonstrating the suitability of this model to analyze, interpret and classify a sample of homicides in Spain.

## Materials and Methods

### Data

The sample consists of 448 cases of homicides carried out in Spain between 2010 and 2012. Of the total, 81% of the homicides were committed by offenders who knew their victim and 11% were carried out by strangers. The majority of the offenders and victims are male (90.8 and 53%, respectively), tend to be Spanish (offenders: 70%; victims: 72%). The mean age of the offenders is 41.16 (SD = 15.18, range = 18–86) and 42.88 for the victims (SD = 19.85, range = 0–94) at the time of the offence.

### Procedure

The database from the Report on Homicides in Spain (*RHS*) 2010–2012 was used (González et al., 2018). It was mobilized and coordinated by the Cabinet of Coordination and Studies of the Secretary of State for Security of Spain, under the Spanish Ministry of the Interior. During the early stages, the parties responsible for this project requested police reports from the corresponding police departments (Civil Guard and National Police forces) and created a database to permit information collection. Next, specialized training was received on how to carry out the data dump procedure and information extraction.

The final RHS database contains a total of 684 homicide cases, and it includes basic characteristics of each homicide, on the sociodemographic background of both victim and offender, and on the offender crime scene behavior.

In accordance with the methodology used in past studies on criminal profiling, only simple homicide cases were included (with an offender and a mortal victim), carried out by individuals over the age of 18 and solved by the police department, reducing the number of analyzed cases to 448.

Then, the variables were selected, referring to past literature on homicides in the field of Investigative Psychology (e.g., Salfati, 2000, 2003; Santtila et al., 2001). Other variables were also included which were not used in prior works, such as, for example, the offender’s escape method and certain sociodemographic characteristics of the victims and offenders that are of special interest since they establish homicide typologies in Spain.

Finally, data purging was performed in order to thoroughly analyze the quality of the information and prepare a matrix for statistical analysis. Variables were dichotomized according to the presence (1) or absence (0) of homicide behavior or characteristics (Canter and Heritage, 1990; Canter and Fritzon, 1998; Gerard et al., 2017).

### Statistical Analysis

The data analysis procedure was carried out in distinct phases. In the first phase, non-metric multidimensional scaling (nMDS) was used, based on R statistic software. The nMDS is a multi-variant exploratory technique that represents the correlations between variables as distances on a bi-dimensional map, in which the proximity between variables indicates the frequency of their collective appearance and therefore, their thematic similarity. This procedure offers a global vision of the relationships between all of the variables which permits analysis and interpretation of the psychological processes underlying the homicide (Salfati and Canter, 1999; Groenen and Borg, 2014). The Jaccard similarity coefficient was used to calculate the association between the variables, since it is the most appropriate for the treatment of data from police sources (Ioannou et al., 2015; Almond et al., 2017b; Hempenstall and Hammond, 2018). Below, the goodness of fit was assessed for the model using Kruskal’s *Stress*-I and the R^2^ coefficient of determination.

In the second phase, the *k-*medoids clustering algorithm was used, with R statistic software in order to establish different homicide typologies. Based on the nMDS coordinates matrix, the variables were grouped in k clusters, using the Manhattan distance. For this, the number of clusters was determined *a priori*, first examining the optimal number of groupings. Next, the internal quality of the groupings was analyzed, considering the Global Silhouette Index and the Dunn index.

In the third and final phase, to assess the suitability of the Action System model, each of the cases was assigned to a dominant functioning cluster or mode. In this way, each case was assigned a score that reflected the proportion of variables present in each cluster, specifically classified in each when the score for this cluster was greater or approximately equal to the sum of the scores of the other three. Cases were considered hybrids when they contained the same proportion of variables in more than two clusters. This classification method based on the proportion of variables has been used in past studies (e.g., Fritzon and Garbutt, 2001; Fritzon and Ridgway, 2001; Häkkänen et al., 2004b).

## Results

### Descriptive Analysis

After performing a univariate analysis of the data, variables that were present in over 70% of the homicides were removed as they occur in the majority of cases and therefore, were not useful when differentiating between cases and identifying themes (Almond et al., 2017a). In addition, variables that occurred in less than 1% were removed because they would have limited utility for classification purpose (Fritzon and Garbutt, 2001).

This study, therefore, used 32 mutually exclusive variables, 19 of which are related to the crime scene, 5 with the offender, 3 with the victim and 5 with the interaction between victim and offender. Table 1 presents a descriptive analysis of the variables used in the subsequent multivariable statistical analysis (see Appendix for the content dictionary).

**TABLE 1:** Descriptive analysis of the variables of the crime scene, offenders and victims.

Variables Name (label)	N (%)
**Crime scene characteristics**	
1. Sharp weapon (sharp)	220 50.6
2. Escape on foot (foot)	120 (39.6)
3. Arrested in crime scene (arrested)	109 (36.8)
4. Method of approach (approach)	106 (36.3)
5. Weapon displacement (wdisplaced)	131 (36.3)
6. Escape by vehicle (vehicleesc)	109 (36)
7. Bring a weapon to crime scene (wbring)	102 (31.7)
8. Crime scene outdoor (outdoor)	135 (30.1)
9. Firearms (firearms)	62 (14.3)
10. Physical force (force)	48 (11)
11. Blunt weapon (blunt)	42 (9.7)
12. Body displacement (bodydispl)	35 (7.9)
13. Hidden body (bodyhidden)	26 (5.9)
14. Property stolen (stole)	18 (5.2)
15. Suffocation (suffocation)	20 (4.6)
16. Crime scene vehicle (csvehicle)	19 (4.2)
17. Staged (staged)	10 (2.3)
18. Offender forensically aware (forensic)	8 (1.8)
19. Sexual assault (sexual)	3 (1)
**Offender characteristics**	
20. Offender aged 31–50 years (off31–50)	225 (50.5)
21. Offenders convicted for crimes against the person (crecord)	145 (43)
22. Non-Spanish offender (non-spanishoff)	134 (30)
23. Suicide (suicide)	70 (15.6)
24. Female offender (femaleoff)	41 (9.2)
**Victim characteristics**	
25. Female victim (femalevic)	209 (46.8)
26. Victim aged 31–50 years (vic31–50)	160 (37.1)
27. Acquaintance (acquaint)	150 (34.5)
28. Intimate relationship (intimate)	136 (31.3)
29. Non-Spanish victim (non-spanishvic)	123 (28)
30. Family (family)	82 (18.9)
31. Stranger (stranger)	39 (9)
32. Other relationship (other)	28 (6.4)

### Multidimensional Scaling

**FIGURE 1:**
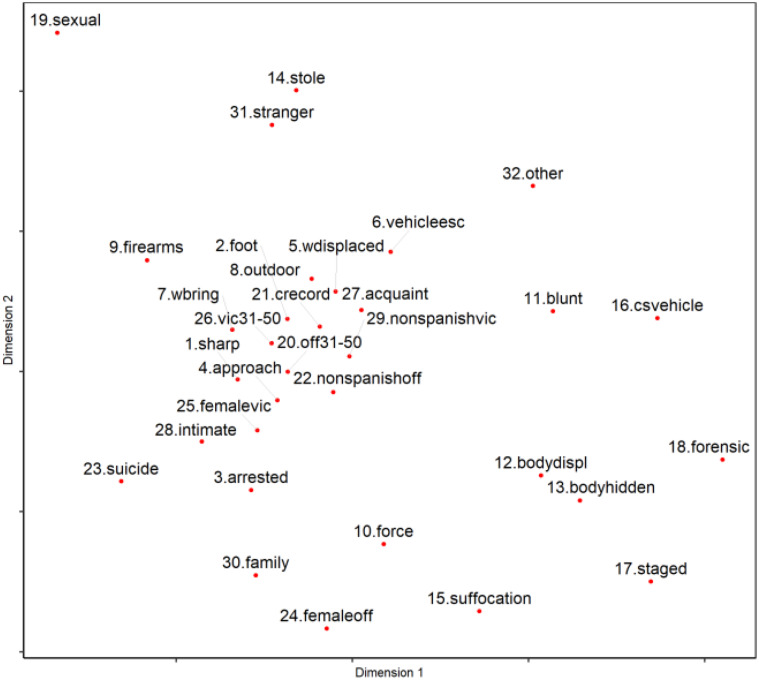
Bi-dimensional map of the nMDS.

Figure 1 presents a bi-dimensional map resulting from the nMDS analysis, which shows the proximity of the 32 variables in a geometric space.

A Stress*-*I index of 0.215 was obtained, suggesting a poor data fit; however, the RSQ is close to 1 (0.795), demonstrating that the fit between disparities and distances is very good. Considering that the stress value is not a conclusive criterion to determine the goodness of fit of the data, it may be assumed that the model has an acceptable goodness of fit. Furthermore, some authors have affirmed that a model may be accepted even when not having a perfect fit, assuming that the spatial representation of the variables permits a significant interpretation (Salfati and Haratsis, 2001; Salfati and Dupont, 2006).

### Cluster Analysis

Even though the solution for five clusters is that suggested by the program, this does not adjust to the theoretical foundations of the Action System model. Therefore, a *k-*medoids clustering algorithm was performed to obtain four groupings.

**FIGURE 2:**
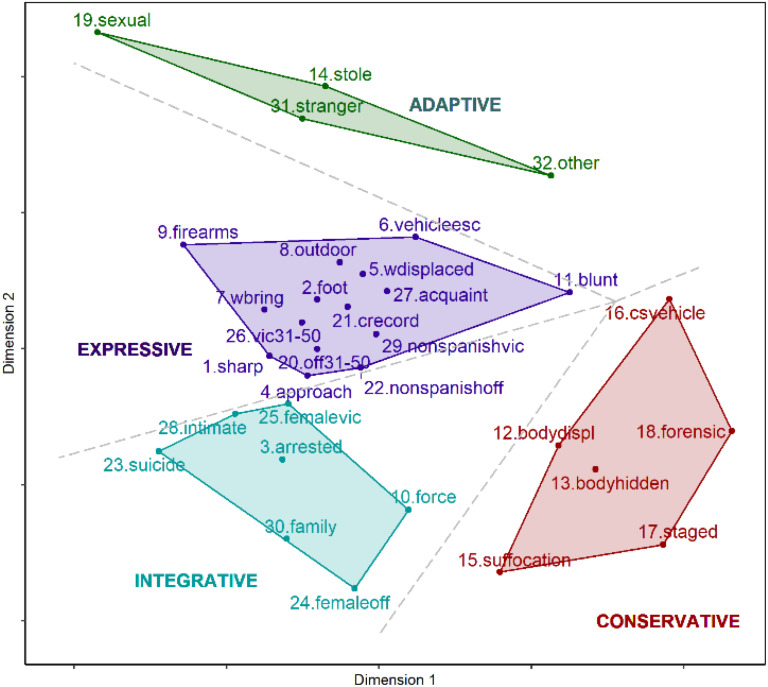
Typology of simple homicides according to the Action System model.

As Figure 2 shows, the behavior of the offenders on the crime scene could be distinguished in terms of internally and externally motivation sources. Some behaviors occurring on the diagonal left-hand side of the plot suggest the expressive form of aggression (internal source of action). Victims tend to be individuals who are known by the offender, making it likely that these are homicides caused by an argument with the victim, with the offender attempting to inflict pain, thus indicating the important role of emotions in these crimes. Also, the offender who committing suicide following the homicide is interpreted as an outward expression of anger which subsequently turned inward. By contrast, the variables located on the vertical right-hand side of the plot (e.g., staged, body hidden, body displaced, sexual, stole) represent actions of an instrumental nature (external source of action) that are the result of an ulterior motive to the violent act.

On the other hand, the behavior of the offenders on the crime scene could also be differentiated in terms of the role of the target. The horizontal top half of the plot reflects the victim the victims are perceived as objects (external target of action) by the aggressor, who has no feelings for them (e.g., stranger, other relationship). Thus, the victims are perceived as a means of achieving the aggressor’s main objective (economic, sexual). In addition, the horizontal bottom half of the plot was suggestive of the victim as person (internal target of action), in that offenders who perceived their victim as significant individuals tend to distance him/herself from the homicide committed (e.g., intimate, family, forensic awareness, body displaced, body hidden).

Therefore, four modes of functioning may be identified which offer a typology of homicides: Conservative, Expressive, Integrative and Adaptive (Figure 2). Likewise, it may be seen that the Expressive and Conservative modes appear in opposition to one another, as is the case with the Adaptive and Integrative modes, with the direction of these latter being perpendicular to that of the Expressive and Conservative modes.

The Global Silhouette obtained a value of 0.37, observing that all of the variables were correctly assigned to the clusters; similarly, the Dunn index had a value of 0.99, therefore there is evidence that the clusters may be correctly separated. Ultimately, the analyses performed to assess the internal quality of the groupings revealed that the clusters had an acceptable internal validity, guaranteeing the goodness of the simple homicide typologies that were established.

#### Expressive Homicides

Cluster 1 is represented in the central part of the plot in Figure 2, and is made up of 15 variables. These are homicides that take place outdoors (outdoor), where the offenders approach their victims with the intent to kill them (approach). To do so, they use blunt objects (blunt), sharp weapons (sharp) and firearms (firearms), which tend to be brought to the scene by the aggressor (wbring) and which, after the homicide, are left at the original site of the event (wdisplaced); in addition, the offenders escape from the crime scene by foot (foot) or by vehicle (vehicleesc). As for the victims, they are between 31 and 50 years old (vic31–50), of foreign nationalities (non-spanishvic), and are known by the aggressors (acquaint). The offenders are between 31 and 50 years old (off31–50), also foreigners (non-spanishoff), and have criminal records for crimes against individuals (crecord). Therefore, taking into account the characteristics of the variables making up this cluster, it is considered that these homicides reflect an Expressive functioning mode.

#### Adaptive Homicides

Cluster 2 is represented in the upper part of the plot in Figure 2. The variables included indicate that these homicides take place during the course of other criminal activities, specifically, robberies (stole) and sexual aggressions (sexual). These homicides are instrumental in nature, since the purpose of the offender is to obtain sexual or economic gratification. The victims are unknown to the offenders (stranger) or have some other type of relationship with them (other). Therefore, these homicides reflect an Adaptive functioning mode.

#### Integrative Homicides

Cluster 3 is represented in the lower part of the plot in Figure 2, and is made up of 7 variables. These homicides take place in the family setting. According to the disposition of the variables in the multi-dimensional space, it has been found that, on the one hand, when the aggressor is female (femaleoff), the victims tend to be individuals from their closet family environment (family), and physical force is the most common homicide method used (force). On the other hand, cases of femicides are found here, in which the victims are women (femalevic) and in a sentimental relationship with the aggressor (intimate). In these latter cases, it is noted that the aggressors tend to be detained at the crime scene or commit suicide after committing the homicide. Given the characteristics of the variables in this cluster, these homicides reflect an Integrative functioning mode.

#### Conservative Homicides

Cluster 4 is represented in the right side of the plot in Figure 2, and is made up of 6 variables. It includes homicides that are characterized by a high degree of criminal planning, since the aggressor acts with a high level of forensic awareness (forensic), not leaving incriminating evidence at the crime scene which could facilitate his/her identification, and often intentionally altering the crime events (staged) to hinder the police investigation. Similarly, the offender often removes the victim’s body from the original site of the crime and/or attempts to hide the body so as to remove all evidence and indica1.tors linking him/her to the homicide (bodydispl, bodyhidden). These homicides tend to take place inside vehicles (csvehicle) and suffocation is the most predominant method used to cause death (suffocation). Therefore, the characteristics of these homicides reflect a Conservative functioning mode.

### Assignment of the Cases to a Dominant Typology

Following a classification criteria based on the proportion of variables, of the 448 homicides, 392 (87.5%) could be assigned to a dominant typology, while 56 (12.2%) cases were classified as hybrids. The majority of the cases were classified in the Expressive mode (52%), followed by the Integrative mode (43.9%). Adaptive represented 2.8% of the homicides and finally, in last place, 1.3% of the cases reflected the Conservative dominant functioning mode. Table 2 shows the results of the classification of the cases.

**TABLE 2:** Classification of cases in a dominant typology.

Typology	N (%)
Expressive homicides	204 (52)
Adaptive homicides	11 (2.8)
Integrative homicides	172 (43.9)
Conservative homicides	5 (1.3)

## Discussion

The results are coherent with past studies that have revealed that the characteristics of homicides and the behaviors carried out by aggressors at the crime scene may be differentiated in terms of functioning mode, facilitating the understanding of the psychological processes underlying homicides (Fritzon and Garbutt, 2001; Fritzon and Brun, 2005; Cullen and Fritzon, 2019). Similarly, the effectiveness and suitability of the Action System model for creating a typology of simple homicides have been demonstrated, establishing connections between the *modus operandi*, victim characteristics and aggressor characteristics that are representative of the studied sample. Four homicide types were identified: Expressive, Conservative, Integrative and Adaptive, and the majority of the cases (87.5%) were classified in a dominant typology.

The Expressive mode was representative of 52% of the cases studied and it included impulsive homicides in which the victims are known by their aggressors. According to Fritzon and Brun (2005), it is likely that this type of violence is the result of an argument between the victim and the offender, although it may also reveal a regular and interiorized way of maintaining interpersonal relations when the aggressor experiences negative feelings, indicating an absence of self-control. However, given the lack of information on emotional states prior to the crime, it was not possible to include variables related to homicide motivations or potential triggers. As for the Expressive offender profile, it is similar to that found in previous studies, tending to refer to offenders with criminal records acting with higher levels of criminal planning and forensic knowledge in order to prevent their identification, thanks to their criminal experience (e.g., displaced weapon, outdoor crime scene, the aggressor escapes from the crime scene) (Cullen and Fritzon, 2019; Pecino-Latorre et al., 2019a).

On the other hand, the Adaptive mode represents 2.8% of the analyzed cases. According to past studies, these homicides are linked to other criminal activities, specifically, robberies and sexual aggressions, reflecting the instrumental nature of the violence used (Fritzon and Brun, 2005). Also, confirming prior studies, the victims do not appear to have any special meaning to the aggressor, and in fact, they are often unknown individuals who are killed for what they may represent (e.g., prostitutes) (Fritzon and Garbutt, 2001; Fritzon and Brun, 2005).

Confirming the results of Fritzon and Garbutt (2001), in the Integrative mode, we find homicides carried out in the family setting, representing 43.9% of the examined sample. This Integrative typology includes two homicide sub-types: (a) female offenders who will a family member (family, female offender) and (b) femicides (female victim, intimate). It is likely that both cases are precipitated by an emotional outburst, such that the violence exercised by the aggressor is directed at the recovery of an emotional balance and alleviation of their feelings of anguish (Fritzon and Garbutt, 2001; Fritzon and Brun, 2005). Femicides are most often characterized by the offender’s subsequent suicide, after committing the homicide, supporting past studies that have demonstrated that the suicide variable is the most representative of this mode (Fritzon and Garbutt, 2001; Fritzon and Brun, 2005; Cullen and Fritzon, 2019). On the other hand, according to Cullen and Fritzon (2019), female offenders were more likely to kill minors from the family environmental, one tentative explanation is that the offenders suffer from an emotional disorder; however, these results may not be confirmed due to the absence of information.

Finally, despite the fact that the Conservative mode represents a minority of the cases (1.3%), it is similar to that which has been found in past studies, highlighting very well-planned homicides in which post mortem actions take place, related to the manipulation of the crime scene, the hiding and the displacement of the body from the original site of the events (Fritzon and Garbutt, 2001; Fritzon and Brun, 2005). According to Fritzon and Brun (2005), this type of violence is instrumental in nature, since the actions are directed at maintaining an integral part of the aggressor’s personality which has been damaged. Similarly, the results support past works in which these homicides have been associated with indirect methods of causing the death of the victim (e.g., suffocation) (Cullen and Fritzon, 2019).

However, it should be noted that past works using the Action System model to establish different homicide profiles have not used a general sample of homicides, but rather, have focused on samples of intra-family and school crimes. Therefore, it is difficult to establish similarities and differences between the results obtained and those found in these studies.

The results of this study should be considered based on the following limitations. First, the lack of information on the homicide motivation and possible triggers of the homicides may prevent a better understanding of the psychological processes underlying the crime. Second, no aggressor profile has been found in relation to the Conservative homicides. This may be because the database lacks sufficient information on homicide characteristics and characteristics related to victims and offenders. Therefore, it may be interesting to complete this database with information from other sources, such as, for example, psychosocial and personality characteristics of the offenders, derived from reports completed by penitentiary institutions. This would help in the creation of more detailed homicide typologies that may establish stronger connections between the *modus operandi* and characteristics of the offenders. Finally, the conclusions derived from this work cannot be generalized to all types of homicides, which limits it is applicability for police investigations.

Future lines of research suggest the replication of this methodology using distinct homicide types (multiple homicides, juvenile homicides). It would also be interesting to discover more about the psychological differences underlying crimes and knowledge of the cognitive processes of the aggressors, given the major practical implications that this could have on police interrogations. For this, future studies should use the Narrative Action System model in order to understand the offender’s interpretations of the homicide, the cognitive distortions that they may have and the motivations that may lead to the criminal act (Canter and Youngs, 2009).

## Conclusion

This work has confirmed the suitability and validity of the Action System model in order to differentiate between the structure of homicides in terms of modes of functioning and to create a typology of simple homicides in Spain, taking into account the relationships between the *modus operandi*, characteristics of victims and characteristics of offenders. This study offers empirical evidence that helps advance prior studies that have used this theoretical framework in distinct homicide samples (Fritzon and Garbutt, 2001; Fritzon and Brun, 2005; Cullen and Fritzon, 2019). Also, this work highlights the utility of theoretical models in interpreting the results that facilitate the understanding of psychological processes underlying homicide.

Similarly, the importance of the close collaboration between police departments and academic and research institutions that promote and study homicide and other crime typologies has been highlighted (González et al., 2018). In this sense, the utility of using large databases with information on the characteristics of the homicides and their participants has been noted. This permits the identification of criminal variations having a certain applied utility.

To conclude, these results have major practical implications on academia, since they offer a general view as to how simple homicides in Spain may be differentiated based on crime scene characteristics, an area that few past studies have considered. Approximately 95% of the cases take place in the family setting and between individuals of a close proximity to the offender, with a minority of homicides taking place between strangers. Also, it has major implications for criminal profiling, specifically, for the creation of more rigorous suspect prioritization methods and improving human resource management and materials used in criminal investigations. In fact, this study has found connections between the four homicide types and distinct offender profiles, potentially of great use in the search for suspects in criminal investigations of simple homicides in Spain. For example, it has been found to be more likely that offenders will be unknown to the victims (strangers) in cases of sexual assaults or homicides taking place during robberies (Adaptive). Likewise, it has been suggested that when the victims are females, there is a greater probability that the offender was her romantic partner or ex-partner (Integrative). And it has been proven highly likely that aggressors will be foreigners, aged 31 and 50, having a criminal record against other individuals, and known to the victim (not strangers), in homicides taking place in outdoor crime scenes and where firearms have been used (Expressive).

## Data Availability Statement

The raw data supporting the conclusions of this article will be made available by the authors, without undue reservation.

## Ethics Statement

Ethical approval was not provided for this study on human participants because the data is from police reports of scenes where there is a deceased victim. This makes it impossible to obtain your informed consent. The data processing has been carried out in accordance with current legislation. Furthermore, the data has been analyzed with the permission and collaboration of the Spanish Ministry of the Interior. Written informed consent for participation was not required for this study in accordance with the national legislation and the institutional requirements.

## Author Contributions

MP-L, RP-H, and MP-F developed the study concept and design. MP-L performed formal analysis and interpretation of data. MP-L and JS-H contributed with data collection. MP-L, JS-H, RP-H, MP-F, and JG wrote, reviewed, and edited the manuscript. All authors approved the final version of the manuscript.

## Conflict of Interest

The authors declare that the research was conducted in the absence of any commercial or financial relationships that could be construed as a potential conflict of interest.

## APPENDIX: CONTENT DICTIONARY

### Crime Scene Characteristics

1. Sharp weapon: A weapon that is made up of a metallic blade or another material having similar physical characteristics, for cutting or puncturing
2. Escape on foot: The aggressor escapes from the crime scene by foot
3. Arrested in crime scene: The aggressor is detained at the crime scene
4. Method of approach: Method of approaching the victim (includes sudden attack, tricking, prior relationship or surprise)
5. Weapon displacement: The weapon was displaced by the aggressor from the crime scene
6. Escape by vehicle: The aggressor uses any type of vehicle to escape from the crime scene
7. Bring a weapon to crime scene: Weapon brought to the crime scene by the aggressor to carry out the act
8. Crime scene outdoor: Homicides carried out in scenes that are exposed to nature
9. Firearms: All portable weapons that have a barrel and that are shot, that are designed to be shot or that may be easily transformed to shoot a pellet, bullet or projectile, by action of a combustible propellant
10. Physical force: The aggressor uses his/her physical force to mortally harm the victim
11. Blunt weapon: An object that lacks a sharp edge and/or blade and that may have dull edges that can be used to hit and cause traumatic injuries
12. Body displacement: The cadaver was displaced from the crime scene
13. Hidden body: The victim’s body was hidden by the aggressor
14. Property stolen: Robbery of objects with physical force, robbery with violence and intimidation and/or robbery and theft of vehicles
15. Suffocation: Refers to actions in which an instrument is used to suffocate the victim
16. Crime scene vehicle: Homicides carried out inside vehicles
17. Staged: Intentional staging of the crime scene by the offender, to mislead the investigation or to make the homicide appear to have been a suicide
18. Offender forensically aware: Existence of forensic knowledge by the aggressor (specialized knowledge permitting the offender to successfully commit the crime or remove evidence)
19. Sexual assault: Sexual assault

### Offender Characteristics

20. Offender aged 31–50 years: Offender is between the age of 31–50 years
21. Offenders convicted for crimes against the person: Record of history of crimes against persons (including homicides)
22. Non-Spanish offender: Non-Spanish offender
23. Suicide: Suicide carried out or attempted by the aggressor (be it at the crime scene or at another location)
24. Female offender

### Victim Characteristics

25. Female victim
26. Victim aged 31–50 years: Victim is between the ages of 31–50
27. Acquaintance: Acquaintance/neighbor, friend, work/commercial, school relations
28. Intimate relationship: Past or present intimate relationship (be it a couple, spouse, ex-couple, separated or divorced)
29. Non-Spanish victim: Non-Spanish victim
30. Family: Victim and offender have some sort of family relationship
31. Stranger: The victim and offender are strangers
32. Other relationship: Had another type of relationship that is not specified in the previous categories
